# Targeted Therapy, Chemotherapy, Immunotherapy and Novel Treatment Options for Different Subtypes of Salivary Gland Cancer

**DOI:** 10.3390/jcm11030720

**Published:** 2022-01-29

**Authors:** Sarina K. Mueller, Marlen Haderlein, Sebastian Lettmaier, Abbas Agaimy, Florian Haller, Markus Hecht, Rainer Fietkau, Heinrich Iro, Konstantinos Mantsopoulos

**Affiliations:** 1 Department of Otolaryngology, Head and Neck Surgery, Friedrich-Alexander-Universität Erlangen-Nürnberg (FAU), 91054 Erlangen, Germany; heinrich.iro@uk-erlangen.de (H.I.); Konstantinos.Mantsopoulos@uk-erlangen.de (K.M.); 2 Working Group Salivary Glands and Thyroid Glands, Germany Otolaryngologic Society, Head and Neck Surgery, 53113 Bonn, Germany; 3 Comprehensive Cancer Center, Interdisciplinary Oncologic Clinic, Friedrich-Alexander-Universität Erlangen-Nürnberg (FAU), 91054 Erlangen, Germany; marlen.haderlein@uk-erlangen.de (M.H.); sebastian.lettmaier@uk-erlangen.de (S.L.); markus.hecht@uk-erlangen.de (M.H.); rainer.fietkau@uk-erlangen.de (R.F.); 4 Department of Radiation Oncology, Friedrich-Alexander-Universität Erlangen-Nürnberg (FAU), 91054 Erlangen, Germany; 5 Department of Pathology, Friedrich-Alexander-Universität Erlangen-Nürnberg (FAU), 91054 Erlangen, Germany; abbas.agaimy@uk-erlangen.de (A.A.); florian.haller@uk-erlangen.de (F.H.)

**Keywords:** immunotherapy, targeted therapy, chemotherapy, salivary gland cancer, adenoid cystic carcinoma, salivary duct carcinoma, mucoepidermoid carcinoma, acinic cell carcinoma

## Abstract

Surgical resection remains the first line treatment for salivary gland cancer (SGC). In the case of locally advanced disease, surgery is followed by adjuvant radiotherapy. Surgical resection should be favored in resectable locoregional recurrent disease as well, and even the complete resection of all distant oligometastases has clinical benefit for the patients. For inoperable and disseminated metastatic disease, a multitude of systemic therapies including chemotherapy, targeted therapy, and immunotherapy are available. In this review, the current therapeutic options for inoperable recurrent or metastatic SGCs are summarized. Systemic treatment can achieve prolonged progression-free and overall survival, while the overall prognosis remains poor. Current clinical trials include only a limited number of patients and mostly combine different histologic subtypes. Additionally, no randomized controlled trial comparing different therapeutic options has been performed. In the future, further studies with a larger patient cohort and ideally only one histologic subtype are needed in order to improve the outcome for SGC patients. However, this may be difficult to accomplish due to the rarity and diversity of the disease. Additionally, molecular analyses need to be performed routinely in order to individualize treatment and to go one step further towards precision medicine.

## 1. Introduction

Salivary gland cancer (SGC) is a rare (0.6–1.4 per 100,000 [1]) and heterogenous group of head and neck tumors. SGCs include more than 20 histological tumor subtypes, according to the 2017 WHO classification of head and neck cancers [2]. SGCs can occur in major and minor salivary glands. The most common histopathologic types include adenoid cystic carcinoma (ACC), mucoepidermoid carcinoma (MEC), acinic cell carcinoma (AcCC), ductal carcinoma (SD), and adenocarcinoma NOS (not otherwise specified). In general, SGCs are classified by their source of origin (major salivary glands versus minor salivary glands) and their histopathologic grading (high grade, intermediate grade, low grade) [3]. Due to their rare occurrence, published studies tend to look at different histopathologic types, grades, and localizations conjointly, diminishing the validity of the data. If the tumor is resectable, surgery will always be the gold standard treatment option for primary and recurrent SGC. For inoperable primary and recurrent SGC as well as oligometastatic SGC different systemic options are available, with varying success rates. This review will summarize the state-of-the-art systemic treatment options for first- and second-line treatment in inoperable primary and recurrent SGC as well as metastatic SGC.

### 1.1. Histopathologic Types: Local Recurrence and Distant Metastasis Rates

#### 1.1.1. Adenoid Cystic Carcinoma

Adenoid cystic carcinomas are relatively rare types of cancer, accounting for 1% of head and neck tumors and 10% of salivary gland tumors [4]. The disease progresses on different timelines, from a relatively slow but relentless growth to rapidly occurring metastases in high-grade disease. The five-year and ten-year patient survival rate is described as about 60% and 50%, respectively. Notably, the twenty-year survival rate is only 20%, due to high incidences of recurrence and metastasis [5]. The lungs constitute the most common site for metastases, while perineural invasion is characteristic of local progression in ACC [6,7,8]. Distant metastasis is the most common type of relapse, with about 30% of patients with distant metastasis not experiencing recurrence of the primary lesion [9,10]. ACCs are characterized by the recurrent gene fusion MYB-NFIB [11], or less commonly, MYBL-NFIB gene fusions [12] or chromosomal rearrangements that juxtapose super-enhancers to the MYB locus and create a positive feedback loop [13]. The grading is defined by histology: Grade I includes tumors with tubular and cribriform areas but without solid components, Grade II includes cribriform tumors that are either pure or mixed with less than 30% solid areas, and Grade III includes tumors with a predominantly solid pattern [14].

#### 1.1.2. Mucoepidermoid Carcinoma

MEC represents the most common primary salivary malignancy, with a prevalence of 16.9% of all salivary gland tumors [15]. In 2003, Tonon et al. [16] described the translocation t (11;19) (q14–21;p12–13) in an MEC patient. The translocation results in a fusion between the genes *CRTC1* (a transcription co-activator), located at chromosomal region 19p13, and *MAML2* (a member of the mastermind-like gene family), located at chromosomal region 11q21 [17]. MECs are divided into high, intermediate, and low grades. High-grade tumors show a five-year disease-specific survival (DSS) of 67%. High-grade MECs are likely to have lymph node metastases at levels I to III (34.0%) [18].

#### 1.1.3. Acinic Cell Carcinoma

Acinic cell carcinoma is commonly considered a low-grade malignant salivary neoplasm. With a ten-year survival of almost 90%, AcCCs possibly have the best prognosis of all SGCs. High-grade transformation in these tumors is a relatively rare event, although it is being increasingly reported [19]. Whereas low-grade tumors represent a good outcome, high-grade AcCCs show a propensity for distant metastases. Distant metastases occur late and are described in the lungs, pleura, brain, and peritoneum and the paraaortic, paratracheal, and mediastinal lymph nodes, as well as cutaneously [20]. Nodal disease occurs in 8–9%, and 75% of the patients present with persistent or recurrent disease [21,22]. Recurrent translocations enabling oncogenic upregulation of transcription factor NR4A3, located at chromosomal region 9q31, represent the molecular genetic hallmark of AciCCs [23,24].

#### 1.1.4. Salivary Duct Carcinoma

Salivary duct carcinomas are rare and represent 1–3% of all malignant salivary gland carcinomas [25]. SDC is a high-grade adenocarcinoma with morphologic and molecular features resembling an invasive ductal carcinoma of the breast. This salivary gland malignancy was originally described by Kleinsasser et al. [26]. Morphologic and molecular features include HER2 gene amplification, mutations of TP53, PIK3CA (Phosphatidylinositol-4,5-Bisphosphate 3-Kinase Catalytic Subunit Alpha), HRAS (Harvey Rat sarcoma virus), and loss or mutation of PTEN (Phosphatase and Tensin homolog). Notably, a recurrent NCOA4-RET fusion has also been found in SDC. A subset of SDC with apocrine morphology is associated with overexpression of androgen receptors [27]. The most widely studied genomic alteration in SDC is copy number gain of *ErbB2*, known as HER-2/*neu* [28]. About 20 to 30% of all SDCs show an amplification of ErbB2. HER2/neu receptor expression is an independent prognostic factor for decreased DFS and DMFS in patients with SDC [29]. Based on the analysis of different molecular markers, tumors can be classified into HER2, luminal androgen receptor-positive, basal-like, luminal, and indeterminate phenotype [30].

Another growth factor commonly overexpressed in SDC is the epidermal growth factor receptor (EGFR) [31]. The majority of SDCs (about 80%) express androgen receptors (ARs) [32]. SDC can metastasize early into regional lymph nodes as well as distant sites. The most common sites of distant disease are the lung and bone, and liver and brain metastases have been reported as well [33]. Additionally, SDCs show a high rate of recurrence [25]. Survival is poor, with most patients surviving only about three years after diagnosis [33].

#### 1.1.5. Adenocarcinoma NOS

Adenocarcinoma NOS is a heterogenous group that accounts for 5–10% of SGCs. Cervical lymph node metastases occur in 23%, whereas distant metastases are more common (37%). The diagnosis is made by excluding any other SGC. The five-year DFS is 57% [34].

## 2. Systemic Therapy for Advanced and Recurrent Disease and Distant Metastases

In the cases of resectable and recurrent locoregional disease and of distant metastatic disease surgical resection is the best option in operable disease regardless of prior treatment [35]. This strategy is recommended in case of a limited number of lung metastases as well, with such patients benefiting from the complete surgical resection of all pulmonary metastases if the pulmonary metastases did not occur prior to 36 months after diagnosis of the primary SGC [36]. For non-surgically resectable tumors, local options including radiotherapy and stereotactic body radiotherapy may be considered [37]. Simultaneous chemotherapy is often used in daily practice due to individual decisions; however, it should only be considered within clinical trials [35,38]. The prognosis for salivary gland carcinomas with distant metastases is poor. Recently, so-called small molecules and targeted therapies (e.g., antibodies against growth factors) have been implemented in the treatment of salivary gland carcinomas. However, there are no randomized controlled trials comparing survival outcomes between different targeted systemic therapy regimens in SGCs. In the following section, therapeutic options are organized by histologic subtype in order to identify preferable options. However, several studies include more than one histologic subtype and are therefore valid for the other histologic subtypes included. Studies investigating targeted therapies are naturally more homogenous compared to studies investigating chemotherapy, which include almost all histologic subtypes. The latter is true for studies investigating immunotherapy as well. Moreover, it is important to consider that the vast majority of studies include more than one anatomical subsite. This is especially important when considering that certain criteria, including tumor size, cannot be projected from the major salivary gland to minor salivary glands [39]. Despite the heterogeneity of the included patients, the sample sizes are generally low, with 10–62 patients included in the phase II studies. Furthermore, there were different endpoints when comparing different studies. Whereas some studies continued until disease progression, unacceptable toxicity, or patient refusal, other studies defined their endpoints as overall response rate (ORR), complete or partial response (CR, PR), stable disease (SD), or disease progression (DP). The majority of studies reported progression-free survival (PFS) and/or overall survival (OS), along with toxicity rates. Additional details are provided in Table 1.

### 2.1. Targeted Therapy and Chemotherapy

#### 2.1.1. Adenoid Cystic Carcinomas

ACCs are known to be difficult to treat and were regarded for decades as resistant to chemotherapy. Through the analysis of molecular characteristics, a multitude of potential therapeutic targets has evolved.

#### 2.1.2. C-Kit and EGFR

As it is known that ACCs have a high c-Kit expression of 90% [40], imatinib was used in several phase II studies [41,72,73]. Imatinib is an inhibitor of c-Kit, bcr-abl and PDGFRA (platelet-derived growth factor receptor alpha); however, it did not show an overall response in ACCs [42]. In combination with cisplatin [74], 10% of the cases showed partial response. The bad ORR (1%) was true for the c-kit, bcr-abl, SRC family, PDGFß (platelet-derived growth factor ß) and EPHA2 inhibitor dasatinib [43]. In ACCs, immunohistological studies showed overexpression of epidermal growth factor receptor (EGFR) [44]. For this reason, phase II studies of cetuximab (EGFR) [45], gefitinib (EGFR) [75], and lapatinib (HER-2, EGFR) [46] have been conducted. For cetuximab, a clinical benefit rate of 50% was described independent of the EGFR expression or copy number gain [45]. However, for all EGFR inhibitors the no ORR was seen.

#### 2.1.3. VEGFR

Further phase II studies targeted angiogenesis in the sense of targeting the vascular endothelial growth factor (VEGFR), as the literature described 76% expression in ACCs. For VEGFR, it was found that there was an association with recurrence and metastases [47]. Medication tested in phase II trials included dovitinib (FGFR, VEGFR, PDGFR, c-Kit) [48,49], sunitinib (VEGFR, c-Kit, PDGFR) [50], regorafenib (VEGRF, FGFR, PDGFR) [51], nintedanib (VEGFR, FGFR, PDGFR) [52], lenvatinib (VEGFR, FGFR, PDGFR) [53,54], axitinib (VEGFR, PDGFR, c-Kit) [55,56], and sorafenib (VEGFR, PDGFR, c-Kit) [62,76]. For sunitinib, regorafenib, and nintedanib, no overall response rates could be seen. For dovitinib, there was an ORR of 3.1% in one study [48].

For sorafenib, axitinib, and lenvatinib, ORRs from 8 to 16% were described. As these targeted therapies represent the best ORRs, those studies will be discussed in more detail. 

Looking first at axitinib, Locati et al., [55] included 26 patients in the study. The ORR was 8% (all PR), the SD was 50%, the DP 42%, the PFS 5.5 months, and the OS 26.2 months. The patients received 10 mg axitinib daily. Adverse advents due to axitinib included stomatitis, fatigue, and hypertension. The study by Ho et al. [56] investigated axitinib and administered 10–20 mg daily in 33 patients. The ORR was 9.1% (again all PR), the SD 75.8%, the DP 12.1%, and the PFS 5.7 months. Both studies show a similarly bad ORR and PFS. Adverse events included hypertension, oral pain, and fatigue. However, the second study showed higher SD and lower DP rates, and performed MYB biomarker analysis and genomic profiling. Consequently, Locati et al. [55] concluded that axitinib is not a treatment option for ACC, whereas Ho et al. [56] suggested that 4q12-amplified ACCs may benefit from tyrosine kinase inhibitor therapy.

Analyzing sorafenib more closely, Thompson et al. [76] looked at 23 ACC patients. This study described an ORR of 11% (all PR), 68% SD, 21% PD, a PFS of 11.3 months, and an OS of 19.6 months. Adverse events included fatigue, weight loss, and hand–foot syndrome, and Grade 3 toxicity was common (57%).

The second study on sorafenib was conducted by Locati et al. [62]. The ORR of the heterogenous group of ACC and non-ACC patients (37 patients) achieved an ORR of 16% (again all partial responses), an SD of 76%, a PFS in the ACC group of 8.9 months, and an OS in the ACC group of 12.3 months. Interestingly, responders included high-grade MEC, SDC and adenocarcinoma NOS. Both studies administered sorafenib 400 mg twice daily.

Looking finally at lenvatinib, Tchekmedyan et al. [53] included 33 ACC patients and used Lenvatinib 24 mg/d. The ORR was 15.6% (only PR), the SD 75%, and the PFS 17.5 months. Adverse events included hypertension and oral pain. Interestingly, 6.3% of the patients discontinued treatment as a result of toxicity before the first scan, 23 patients required at least one dose modification, and 56.3% of the patients discontinued lenvatinib for drug-related reasons. Grade 4 adverse events included myocardial infarction, posterior reversible encephalopathy syndrome, and intracranial hemorrhage.

Another phase II trial by Locati et al. [54] described modest activity with manageable toxicity for Lenvatinib (28 ACC patients). The ORR was 11.5% (again all PR), the PFS 9.1 months, and the OS 27 months. Adverse events included asthenia, hypertension, and decreased weight. In this study, adverse events were again frequent (96% total with Grade ≥ 3 in 50%), and again a dose reduction was necessary in 85.7% of patients due to adverse events. 

To summarize, sorafenib and lenvatinib show the highest ORRs for all tyrosine kinase inhibitors (sorafenib 11% and 16% respectively; lenvatinib 11.5% and 15.6% respectively). While the ORRs remain low, SD rates range between 68% and 76%. Toxicities should not be ignored, and according to these studies this may be especially true for lenvatinib. However, the authors concluded that the toxicity was comparable to previous studies and that patients need monitoring and early management. 

#### 2.1.4. Other Targets

There have been a multitude of targets in phase I and phase II studies that did not show a response to medication. For those targets, see the review of Sahara et al. [42] for details.

#### 2.1.5. Single and Combination Cytotoxic Chemotherapy

Concerning single or combination cytotoxic chemotherapies, prospective evaluation has been limited by small patient numbers, the inclusion of heterogenous populations, and the lack of control groups [35]. As a result, there is no high-level evidence indicating a survival benefit for cytotoxic chemotherapy. However, a small percentage of patients do respond, suggesting potential for these therapies in reducing the tumor burden and burden-related symptom load. The amount of chemotherapy cycles per study ranges between four and six cycles. 

There are two phase II trials by Licitra et al. [61,63] analyzing cisplatin 100 mg/m^2^ as monotherapy (once every 21 days) or in combination with doxorubicin and cyclophosphamide (CAP scheme: cyclophosphamide 500 mg/m^2^, doxorubicin 80 mg/m^2^, cisplatin 80 mg/m^2^). The first study achieved an overall response rate of 18% and an OS of 14 months. The second study analyzed a heterogenous group consisting of ACCs, myoepitheliomas, SDCs, adenocarcinomas, MECs, neuroendocrine tumors, and undifferentiated tumors (22 patients in total). The study achieved an ORR of 27% (all PR) and an OS of 21 months. Adverse events included neutropenia and stomatitis. 

Debaere et al. [64] reported on the CAP scheme in a cohort of ACCs and adenocarcinomas (15 patients). In this study, an ORR of 60% was reached, leading to a PFS of 6.6 months and an OS of 15.1 months. Adverse events included neutropenia, neutropenic fever, and alopecia. With ORRs of 27–60%, the CAP scheme may be considered as the chemotherapy of first choice. However, one has to keep the high toxicity levels in mind, which should always be considered.

Laurie et al. [66] explored the combination of cisplatin and gemcitabine in a heterogenous cohort of 33 patients (ACC, adenocarcinoma, MEC, other). They used cisplatin 80 mg/m^2^ once or carboplatin at an AUC (area under the curve) of five once, as well as gemcitabine 1000 mg/m^2^ on days one and eight of a three-week cycle. The ORR was 24%. These responses were described in ACCs, adenocarcinomas, MECs and SDCs. Adverse events included hearing loss, fatigue, and nausea. For this study, the authors concluded that the study did not achieve its endpoints. However, chemotherapy with cisplatin as single therapy or in combination with doxorubicin and cyclophosphamide may be an option for inoperable recurrent and/or metastatic SGC, while always considering its toxicity. Whether chemotherapies or targeted therapies are more favorable in ACCs cannot be determined, as the studies investigating chemotherapies which show higher ORRs include a heterogenous patient group that may be responsible for the better outcome. 

Final remarks: The interpretation of the meaning of ORRs and the percentage of SD is difficult, mainly because a differentiation has to be made between stable disease due to slow tumor progress and stability due to a medication effect. The VEGFR inhibitors sorafenib, axitinib, and lenvatinib and cytotoxic chemotherapy with platinum ± doxorubicin/cyclophosphamide seem to show the best response rates. While the EGFR inhibitor cetuximab was not convincing in the described study, it may, however, be used as it shows benefits in a clinical setting (Table 2).

### 2.2. Mucoepidermoid Carcinoma

There has been no phase II study that exclusively looked at MECs. Targeted therapies investigating MECs included cetuximab [45], nintedanib [52], and sorafenib [62], with the results shown in the ACC section. There has been no subgroup analysis for different histologic subtypes. Concerning chemotherapy, MECs were included in the trials of the CAP scheme [61], cisplatin and gemcitabine [66], cisplatin and vinorelbine [65,67], and paclitaxel [77], with reasonable results. Thus, these chemotherapies may be used for MEC patients. Other targets, including histone deacetylase 7 [78], are being investigated in vitro and may lead to novel therapeutic approaches for MECs.

### 2.3. Acinic Cell Carcinomas

There has been no phase II study exclusively looking at AcCCs. As with MECs, AcCCs were included in the study analyzing cetuximab [45] and nintedanib [52]. For recurrent or metastatic AcCCs, no specific substance has yet been identified. Although mTOR inhibitors are being discussed [79], no phase I/II trial has yet been performed. Platinum and cetuximab [45] are commonly used in clinical settings.

### 2.4. Salivary Duct Carcinoma

Targets of SDCs include mainly ErbB2/HER-2 and the androgen receptor. Depending on the expression, different medication can be used.

#### 2.4.1. ErbB2/HER-2

Trastuzumab, an inhibitor of ErbB2/HER-2, has shown promising responses as a single therapy, or in combination with taxanes in some case reports [80,81]. Several other single-case reports have been published in combination with platinum, as summarized by Keller et al. [57], showing complete and/or prolonged responses in patients with ErbB2-positive recurrent or metastatic SDC. 

One phase II study by Takahashi et al. [82] administered trastuzumab and docetaxel (trastuzumab 6–8 mg/kg, docetaxel 70 mg/m^2^) in 57 EGFR-positive SDCs. The study showed an ORR of 70.2%, a PFS of 8.9 months, and an OS of 39.7 months. However, adverse events were frequently seen (grade ≥ 3.94%). Dual Erb2 blockade therapy with the combination of a trastuzumab-based regimen either with pertuzumab [83] or lapatinib [84] was shown to have a survival benefit in breast cancer patients. Projecting these results to SDCs, one preliminary study in seven patients using trastuzumab and pertuzumab in Erb2-activated SGCs showed an ORR of 71.4%, which warrants further investigation [85]. In breast cancer, the expression of ErbB2/HER-2 is predictive of the response rate of an inhibitor. For SDCs, more studies are needed to validate those findings and find predictive biomarkers [33].

Moreover, there are case reports showing response after progression on trastuzumab with new drugs such as ado-trastuzumab-emtansine (e.g., Second-Line Treatment of HER2-Positive SGC: Ado-Trastuzumab Emtansine (T-DM1) after Progression on Trastuzumab, [86]) or trastuzumab-deruxtecan (Targeting HER2 with Trastuzumab Deruxtecan, [87]). Moreover, there are more and more retrospective studies showing a benefit of adjuvant trastuzumab in SGC [88].

#### 2.4.2. Androgen Receptor Inhibitors/GnRH/LHRH Agonists

For androgen receptor (AR) antagonists, there are several single case reports showing complete and/or prolonged responses in patients with recurrent and/or metastatic SDC; for more details, see the reviews by Keller et al. [57] and Dalin et al. [58]. AR inhibitors are often used in combination with GnRH/LHRH agonists (triptorelin or goserelin), which inhibit the production of androgens [33].

One phase II clinical trial by Fushimi et al. [59] included 36 patients with AR-positive adenocarcinomas and SDCs treated with a dual AR blockade (leuprorelin, bicalutamide). Leuprorelin acetate was administered subcutaneously at a dose of 3.75 mg every four weeks. Bicalutamide was administered orally at 80 mg daily. The ORR was 41.7%, the PFS was 8.8 months, and the OS was 30.5 months. Adverse events included elevation of liver transaminases and increased serum creatinine. A discontinuation of the dual AR blockage was only necessary in one patient. Consequently, this therapeutic regimen may be useful in AR-positive SGCs with a manageable toxicity level.

Locati et al., 2021 [60] performed a phase II study administering abiraterone 1 g daily plus prednisone 10 mg and luteinizing hormone-releasing hormone in 24 AR-positive SDCs. The ORR was 21%, with 5% partial responses. The PFS was 3.65 months and the OS was 22.5 months. Adverse events included fatigue, flushing, and tachycardia. Adverse events were frequent (92%) at Grade 3 in 25%, however, there were no adverse events at Grade > 3. Thus, this combination regimen may be used as a second-line option in AR-expressing, castration-resistant SGC.

Ho et al. [89] investigated 46 patients with AR-positive SGC and prior AR-targeted therapy in a phase II study of enzalutamide (160 mg). The study described 15.2% ORR (all PR) and 42.2% with SD. Further data are needed to evaluate the clinical benefit for the patients.

#### 2.4.3. Other Targets

Other agents, including lapatinib (ErbB2/HER-2, EGFR), sorafenib (VEGFR, PDGFR, c-Kit) [62], (VEGFR, FGFR, PDGFR) [52], and vemurafenib (BRAF) did not show a response. Details about all studies can be found in the review by Uijen et al. [68].

Final remarks: For ErbB2/HER-2-positive SGC/SDC, the combination of trastuzumab and docetaxel may be used. For AR-positive SGC/SDC, dual AR blockage with leuprorelin and bicalutamide may be used. As a second line treatment, the combined regimen of abiraterone, prednisolone, and luteinizing hormone-releasing hormone may be used. SDCs were included in the study of Licitra et al. [61] investigating the CAP scheme. Therefore, single or combined schemes including cisplatin may be used as well (Table 3).

### 2.5. Adenocarcinoma NOS

There has not yet been a study focusing solely on systemic therapies for adenocarcinomas NOS. However, adenocarcinomas were included in an investigation of nintedanib [52], sorafenib [62], cetuximab [45], and leuprorelin/bicalutamide [59]. Moreover, adenocarcinomas were included in six chemotherapy studies [61,63,65,66,77,90], which are displayed below in detail. Whereas cisplatin monotherapy and the CAP scheme have already been presented in the ACC section, the other four studies will be discussed in detail below. However, it must again be noted that all of these studies included diverse histological subtypes, not only adenocarcinomas (NOS).

#### Vinca Alkaloids/Platinum

One phase II trial by Airoldi et al. [67] compared 16 patients receiving cisplatin 80 mg/m^2^ on day one plus vinorelbine (VNB) 25 mg/m^2^ on days one and eight (every three weeks) for a minimum of three cycles, with 20 patients receiving VNB 30 mg/m^2^/week for a minimum of nine weeks. Adenocarcinomas, ACCs and MEC were included. The ORR of the combined group was 44% (19% complete responses), and the ORR of the VNB mono group was 20% (0% complete responses). SD was seen in 37.5% and 45%, respectively, while PD was noted in 19% and 35%. Adverse events including nausea and emesis were found to be significantly higher in the combined group for both side effects. However, there were no differences regarding other side effects.

Hong et al. [65] analyzed the combination of vinorelbine 25 mg/m^2^ on days one and eight plus cisplatin 80 mg/m^2^ on day one every three weeks for four or six cycles. This study included patients with ACC, adenocarcinoma, AcCC, MMEC, undifferentiated carcinoma and carcinoma ex pleomorphic adenoma. The ORR was 1%, 33% of the patients had SD, and 62% had PD. The PFS was 6.3 months and the OS 16.9 months. Adverse events included anemia, leucopenia, and neutropenia.

Airoldi et al. [90] used the combination of vinorelbine 25 mg/m^2^ on days one and eight plus cisplatin 80 mg/m^2^ on day one every three weeks for a maximum of six cycles (median five cycles) in 60 patients. This study included adenocarcinomas and undifferentiated carcinomas. The ORR after five cycles was 51.7%, with 7% complete responses and 24% partial responses; 33% had SD and 36% had PD. For four cycles, the ORR was only 5% (only partial responses). Interestingly, the best ORR was observed in adenocarcinomas (54%). However, there were undifferentiated carcinomas in the group the ORR was compared to.

To summarize, cisplatin or the combination of vinorelbine and cisplatin may be considered for SGC patients. Especially for first-line chemotherapy in adenocarcinomas, it may show a benefit, whereas for second-line chemotherapy it shows only low palliative activity. Vinorelbine as a monotherapy shows worse ORRs.

A study by Gilbert et al. [77] investigated paclitaxel 200 mg/m^2^ every three weeks for a minimum of four cycles in 45 patients with ACC, adenocarcinomas and MECs. The ORR was 26% (all partial responses). All responses were seen in adenocarcinomas and MECs, and no responses were seen in the ACCs. For OS, however, there were no differences between the histologic subtypes. Adverse events included leucopenia, granulocytopenia and infection.

Final remarks: Based on these studies, chemotherapy including cisplatin, CAP, cisplatin/vinorelbine and paclitaxel may be used in treatment of adenocarcinomas (Table 4).

## 3. Immunotherapy

### 3.1. In Vitro

After the Nobel Prize was awarded to James Allison and Tasuku Honjo in 2018, immune checkpoint inhibitors targeting PD-1/PD-L1 (Programmed cell death protein 1/Programmed cell death 1 ligand 1) and CTLA-4 (cytotoxic T-lymphocyte-associated Protein 4) have been implemented in the treatment of the majority of solid tumors. 

First, in vitro studies have been conducted which assessed the PD-L1 status of different subtypes. In SDCs, more than half of the specimens expressed PD-L1, thereby suggesting that anti-PD-1/PD-L1 or other checkpoint inhibitors may have some anti-tumor activity in this disease [91]. Another study analyzed CTLA-4 cells and noted that the positive count was high (83% and 90% respectively). The density of CTLA-4+ cells, however, was described as less than PD-1+ cells [92]. For ACCs, there was low immunogenic activity, showing low CD8^+^, GrB^+^ tumor-infiltrating lymphocytes, CD1a, and CD83 populations as well as scarce positivity for CTLA-4 and PD-1 [93]. For AcCCs, a higher number of tumor-infiltrating T- and B-lymphocytes was significantly associated with high-grade transformation. Furthermore, higher counts of T-lymphocytes correlated with node-positive disease. There was a significant correlation of higher levels of PD-L1 expression and lymph node metastases with the occurrence of high-grade transformation. Moreover, PD-L1 CPS was associated with a poor prognosis regarding metastasis-free survival. The authors concluded that increased immune cell infiltration of T and B cells as well as higher levels of PD-L1 expression in AcCC in association with high-grade transformation, lymph node metastasis, and an unfavorable prognosis suggests a relevant interaction between tumor cells and immune cell infiltrates in a subset of AcCCs, and might represent a rationale for immune checkpoint inhibition [94]. Finally, a study analyzing all histological subtypes including MECs and adenocarcinomas described significantly elevated CD3+, tumor proportion scores (TPS), combined positive scores (CPS), and immune cell scores (IC) in adenocarcinoma NOS compared to ACCs, MECs and AcCCs. CPS was correlated with node-positive disease; moreover, adenocarcinoma NOS displayed IC scores of 2 or 3 in the majority of cases, and was associated with a poor prognosis for PFS and OS. CPS correlated with strong nuclear or null p53 staining in adenocarcinoma NOS and not in other SGCs. Long-lasting partial remission was achieved in one adenocarcinoma NOS patient who received Pembrolizumab as third-line therapy [69].

### 3.2. Clinical Trials

Despite the recent advances in PD-1 and PDL-1 inhibitor therapies, few clinical trials have been performed. The phase II study of Rodriguez et al. [70] included 25 patients with SGCs (including ACC, AcCC, MEC, adenocarcinoma, SDC) with progressing incurable disease. The treatment regimen included pembrolizumab 200 mg intravenously every three weeks and the histone deacetylase inhibitor vorinostat 400 mg orally five days on and two days off during each 21-day cycle. The ORR was 4% (all partial responses), the PFS 6.9 months, and the OS 14 months. These results are comparable to the worst outcomes with targeted therapies in ACCs. Adverse events included renal insufficiency, fatigue, and nausea, and 36% of the patients experienced adverse events Grade ≥ 3. Three deaths were reported. However, the authors mentioned that toxicity levels were not higher than for other cancers in the head and neck area or for pembrolizumab alone. The ORR was noted to be less in SGC compared to other head and neck areas.

Mahmood et al. [71] conducted a small randomized phase II study in 20 patients with progressing ACC, comparing single-agent pembrolizumab (200 mg intravenously every three weeks) and pembrolizumab plus hypofractionated radiations (30 Gy in five fractions) to a site of metastatic disease. The ORR was 0, and 60% had SD. In the pembrolizumab group, the PFS and OS were 0 and 27.2 months, respectively. In the pembrolizumab plus radiation group, the PFS and OS were 4.5 months and 6.6 months, respectively. Adverse events included liver enzyme activation. There was no ORR for ACCs, which seem to be non-responsive to immunotherapy. Here, again, the SD is difficult to interpret as disease may progress slowly without the effect necessarily being linked to pembrolizumab.

The Keynote-028 study [95], a phase Ib study, investigated pembrolizumab in a cohort of 26 patients. PD-L1-positive adenocarcinomas, undifferentiated carcinomas, MECs, squamous cell carcinomas, and ACCs were included. Patients received pembrolizumab 10 mg/kg every two weeks for ≥2 years, or until confirmed disease progression or unacceptable toxicity. The ORR was 12% (all partial responses). The median duration of response was four months. Adverse events included diarrhea, decreased appetite, pruritus, and fatigue. One patient died of interstitial lung disease.

Final remarks: Immunotherapy with pembrolizumab did not show the same ORRs in SGCs as are known from other head and neck subsites. However, the Keynote-028 study describes response rates of up to 12%, which may be promising for some histological subtypes. As these studies only included a small sample size and a very heterogenous collective, clinical effectiveness on different histological subtypes remains elusive. The effect on ACCs seems to be non-existent at the present time. Studies with a larger sample size and a more homogenous collective are needed in the future.

## 4. Potential New Systemic Therapeutic Strategies

In a prospective phase II study, 93% (13 out of 14 patients) of ACC patients and 40% (4 out of 10 patients) of patients with SDC showed a relevant PSMA radionuclide uptake in 68GA-PSMA-PET-CT. These patients might benefit from PSMA radionuclide therapy. Studies investigating Lutetium-177-PSMA Radioligand Therapy (known from prostate cancer) in SGC are ongoing (NCT04291300) [96].

Other interesting ongoing trials are investigating targeted therapy against MYB (in ACC), NOTCH, and c-MET. Moreover, phase II studies investigating double checkpoint inhibition (PD-1/PD-L1 and CTLA-4) and PD-1/PD-L1 inhibitors in combination with VEGFR antibodies are recruiting patients [97].

## 5. Targeted Therapies Based on Actionable Molecular Alterations

Finally, targeted therapies based on actionable molecular alterations are one promising approach for individualized therapeutic management and precision medicine. All patients should be screened for a defined panel of driver mutations. Driver mutation-negative patients should be offered target-specific testing [35,98]. Preliminary studies [99] show promising results which will need to be validated in the future.

## 6. Conclusions

Systemic therapy for SGC should include targeted therapy if tumor-specific targets are available. Chemotherapy may be considered for all histologic subtypes independent of molecular targets. The clinical benefit of immunotherapies will have to be evaluated in future studies. In general, PFS and OS are normally low for inoperable recurrent or metastatic SGCs. However, current clinical trials include rather small sample sizes and frequently heterogenous patient collectives due to the rarity and diversity of the disease. This inadequacy urgently needs to be remedied in future studies. Additionally, molecular analyses need to be performed routinely in order to individualize therapies and continue to advance towards precision medicine.

## Figures and Tables

**Table 1:** Summary of all targeted therapies, chemotherapies, and immunotherapies.

Study	Therapy	Dose *	Target	Histological Subtypes	Sample Size **	ORR (%)	Complete Response (%)	Partial Response (%)	SD (%)	DP (%)	PFS (Months)	OS (Months)	Toxicity (Most Common)
**Phase II**	**Targeted Therapy**												
Pfeffer et al. [39]	imatinib	300–800 mg/d	c-kit, bcr-abl, PDGRF	c-kit positive ACC	10	0	0	0	2				diarrhea, fatigue, periorbital edema
Hotte et al. [40]	imatinib	600–800 mg/d	c-kit, bcr-abl, PDGRF	c-kit positive ACC	16	0	0	0	6	6	7.5		rash, headache, dyspnea
Ghosal et al. [41]	imatinib, cisplatin	imatinib 400–800 mg/d, cisplatin 80 mg/m^2^	c-kit, bcr-abl, PDGRF	c-kit positive ACC	28	10.3	0	10.3	67.9		15	35	anemia, thrombocytopenia, fatigue, facial edema
Wong et al. [42]	dasatinib	100 mg/d	the c-kit, bcr-abl, SRC family, PDGFß, EPHA2	c-it posiitive ACC, non-ACC	54	1/0	0	0	48.8/53.8	70.7/30.8	4.8		fatigue, nausea, headache
Locati et al. [43]	cetuximab	200–400 mg/m^2^	EGFR	ACC, MEC, myoepihelial, AcCC, cystadenocarcinoma	30	0	0	0	80				skin rash, pruritus, hair loss
Jakob et al. [44]	gefitinib	250 mg/d	EGFR	ACC, non-ACC	37	0	0	0		91.7	4.3/2.1	25.9/16	diarrhea, rash, fatigue
Agulnik et al. [45]	lapatinib	1500 mg/d	HER-2, EGFR	EGFR/HER-2 positive ACC, non-ACC	62	0	0	0	79/47	21/53			diarrhea, fatigue, rash
Keam et al. [46]	dovtinib	500 mg/d	VEGFR, FGFR, PDGFR, c-kit	ACC	32	3.1	0	3.1	93.8		6		asthenia, myalgia, diarrhea
Dillon et al. [47]	dovtinib	500 mg/d	VEGFR, FGFR, PDGFR, c-kit	ACC	35	0	0	6	65		8.2	20.6	fatigue, anorexia, nausea
Chau et al. [48]	sunitinib	37.5 mg/d	VEGFR, c-kit, PDGFR	ACC	14	0	0	0	78.6			18.7	fatigue, oral mucositis, hypophosphatemia
Ho et al. [49]	regorafenib	120–160 mg/d	VEGFR, FGFR, PDGFR	ACC	38	0	0	0	42				
Kim at al. [50]	nintedanib	400 mg (200 mg twice daily)	VEGFR, FGFR, PDGFR	ACC, adenocarcinoma, MEC, SDC, AcCC	20	0	0	0					Liver enzyme elevation, nausea
Tchekmedyan et al. [51]	lenvatinib	24 mg/d	VEGFR, FGFR, PDGFR	ACC	33	15.6	0	15.6	75		17.5		hypertension, oral pain
Locati et al. [52]	lenvatinib	24 mg/d	VEGFR, FGFR, PDGFR	ACC	28	11.5	0	11.5			9.1	27	asthenia, hypertension, decreased weight
Locati et al. [53]	axitinib	10 mg/d	VEGFR, PDGFR, c-Kit	ACC, non-ACC	26	8	0	8	50	42	5.5	26.2	stomatitis, fatigue, hypertension
Ho et al. [54]	axitinib	10 mg–20 mg/d	VEGFR, PDGFR, c-Kit	ACC, non-ACC	33	9.1	0	9.1	75.8	12.1	5.7		hypertension, oral pain, fatigue
Thomson et al. [55]	sorafenib	800 mg/d	VEGFR, PDGFR, c-Kit	ACC	23	11	0	11	68	21	11.3	19.6	fatigue, weight loss, hand foot syndrome
Locati et al. [56]	sorafenib	800 mg/d	VEGFR, PDGFR, c-Kit	ACC, non-ACC (adenocarcinoma, SDC, MEC)	37	16	0	16.2	76		8.9/4.2	26.4/12.3	
Takahashi et al. [57]	trastuzumab, docetaxel	trastuzumab 6–8 mg/kg, docetaxel 70 mg/m^2^, q d22	ErbB2/HER-2	EGFR-positive SDC	57	70.2					8.9	39.7	anemia, decreased EBC, neutropenia
Fushimi et al. [58]	leuprorelin, bicalutamide	leuprorelin 3.75 mg, bicalutamide 80 mg	dual androgen-receptor	AR-positive adenocarcinoma, SDC	36	41.7					8.8	30.5	elevated liver transaminases, increased serum creatinine
Locati et al. [59]	abiraterone	1 g (plus prednisolone 10 mg, luteinizing hormone-releasing hormone)	androgen-receptor (CYP17A1)	AR-positive SDC	24	21		5			3.65	22.5	fatigue, flushing, tachycardia
Ho et al. [60]	enzalutamide	160 mg/d	androgen-receptor	AR-positive SDC	46	15.2	0	15.2	42.2		5.5		
	**Chemotherapy**												
Licitra et al. [61]	cisplatin	100 mg/m^2^, q d22				18						14	
Licitra et al. [62]	cyclophophamide, doxorubicin, cisplatin (CAP)	cyclophosphamide 500 mg/m^2^, doxorubicin 80 mg/m^2^, cisplatin 80 mg/m^2^, q d28		ACC, myoepithelioma, SDC, adenocarcinoma, MEC, NEC, undiff.	22	27	0	27				21	neutropenia, stomatitis
Debaere et al. [63]	cyclophophamide, doxorubicin, cisplatin (CAP)			ACC, adenocarcinoma	15	60					6.6	15.1	neutropenia, neutropenic fever, alopecia
Laurie et al. [64]	cisplatin, gemcitabine	cisplatin 70 mg/m^2^ or carboplatin AUC 5 d2, gemcitabine 1000 mg/m^2^ d1.8, q d22		ACC, adenocarcinoma, MEC, other	33	24							nausea, fatigue, hearing loss
Gilbert et al. [65]	paclitaxel	200 mg/m^2^ q d22		ACC, adenocarcinoma, MEC	45	26	0	26					leucopenia, granulocytopenia, infection
Airoldi et al. [66]	cisplatin plus vinorelbin vs. vinorelbin	vinorelbin 25 mg/m^2^ d1 and d8, cisplatin 80 mg/m^2^ d1 vs. vinorelbin 30 mg/m^2^ weekly		ACC, MEC, adenocarcinoma	36	44/20	19/0	25/20	37.5/45	19/35			nausea
Hong et al. [67]	vinorelbin, cisplatin	vinorelbin 25 mg/m^2^ d1 and d8, cisplatin 80 mg/m^2^ d1, q d22		ACC, adenocarcinoma, AcCC, MEC, undiff., carcinoma ex pleomorphic adenoma	40	1	1	0	33	62	6.3	16.9	anemia, leucopenia, neutropenia
Airoldi et al. [68]	vinorelbin, cisplatin	vinorelbin 25 mg/m^2^ d1 and d8, cisplatin 80 mg/m^2^ d1, q d22		adenocarcinoma, undifferentiated	60	51.7	7	24				10	
	**Immunotherapy**												
Rodriguez et al. [69] (phase II)	pembrolizumab, vorinostat	pembrolizumab 200 mg fixed dose every 3 weeks, vorinostat 400 mg 5 days on, 2 days off	PD-1, histone deacetylase	ACC, AcCC, MEC, adenocarcinoma, SDC	25	4	0	4			6.9	14	renal insufficiency, fatigue, nausea
Mahmood et al. [70] (phase II)	pembrolizumab vs pembrolizumab, RT	200 mg fixed dose every 3 weeks	PD-1	ACC	20	0	0	0	60		0/4.5	27.2/6.6	liver enzyme elevation
Cohen et al. [71], Keynote-028 (phase Ib)	pembrolizumab	10 mg/kg every 3 weeks	PD-1	PD-L1-positive adenocarcinoma, undifferentiated, MEC, squamous cell ACC	26	12	0	11.5					diarrhea, pruritus, fatigue

In detail, the table contains the first author, citation, therapy and dosage, target, histological subtypes, sample size, and the outcome measures for overall response rate (ORR), complete responses (%), partial responses (%), stable disease (SD), disease progression (DP), progression-free survival (PFS), overall survival (OS), and the most common toxicities. * The column dose contains only the administered dose and not the number of cycles ** The sample size includes all included patients, even if some studies did not administer medication to all included patients.

**Table 2:** Current therapeutic options for ACCs. The most promising therapeutic options according to literature are shown in bold.

Therapy
Targeted Therapy
imatinib
imatinib, cisplatin
dasatinib
**cetuximab**
gefitinib
lapatinib
dovtinib
sunitinib
regorafenib
nintedanib
**lenvatinib**
**axitinib**
**sorafenib**
**chemotherapy**
**cisplatin**
**cyclophophamide, doxorubicin, cisplatin (CAP)**
cisplatin, gemcitabine
paclitaxel
cisplatin plus vinorelbin vs. vinorelbin
vinorelbin, cisplatin
**Immunotherapy**
pembrolizumab, vorinostat
pembrolizumab vs. pembrolizumab, RT
pembrolizumab

**Table 3:** Current therapeutic options for SDCs. The most promising therapeutic options according to literature are shown in bold.

Therapy
Targeted Therapy
nintedanib
sorafenib
**trastuzumab, docetaxel**
**leuprorelin, bicalutamide**
**abiraterone**
**enzalutamide**
**chemotherapy**
**cisplatin**
**cyclophophamide, doxorubicin, cisplatin (CAP)**
**Immunotherapy**
pembrolizumab, vorinostat

**Table 4:** Current therapeutic options for Adenocarcinoma NOS. The most promising therapeutic options according to literature are shown in bold.

Therapy
Targeted Therapy
cetuximab
nintedanib
sorafenib
leuprorelin, bicalutamide
**chemotherapy**
**cisplatin**
**cyclophophamide, doxorubicin, cisplatin (CAP)**
**cisplatin, gemcitabine**
**paclitaxel**
**vinorelbin, cisplatin**
**Immunotherapy**
pembrolizumab, vorinostat
pembrolizumab

## Abbreviations

AcCC	acinic cell carcinoma
ACC	adenoid cystic carcinoma
AR	androgen-receptor
EGFR	epidermal growth factor receptor
FGFR	fibroblast growth factor receptor
HER-2	human epidermal growth factor receptor 2
MEC	mucoepidermoid carcinoma
PD-1	programmed death receptor 1
PD-L1	programmed death receptor ligand 1
PDGRF	platelet derived growth factor receptor beta
SDC	salivary duct carcinoma
VEGF	vascular endothelial growth factor
